# Physical Education and Improvement

**Published:** 1905-07-22

**Authors:** 


### Physical Education and Improvement.

The National League for Physical Education and Improvement, as constituted by the great meeting at the Mansion House on June 28, has now decided upon the foregoing as its precise title, and has put forth its manifesto asking for members and for pecuniary support. It proposes to establish the two grades of Fellow and Member, a life Fellowship being attainable by a single donation of ten guineas, while ordinary Fellows will pay a guinea, and Members five shillings, annually. Besides these subscriptions, it desires to receive donations, or subscriptions in excess of the qualifying amounts, from any who may be disposed to lend such aid to its proceedings. Its programme is very extensive, and may at present be said to err somewhat on the side of indefiniteness. It does not seek to displace any agencies which are now working for the national welfare, but to bring those agencies into co-ordination and co-operation, to amend where necessary and to extend existing methods of working and, above all, "to promote a patriotic and intelligent public opinion upon this great national question." The programme goes on to say that the League will not concern itself with profitless controversy, that it will not debate the question whether men are weaker to-day than formerly, but will strive to make them stronger. It recognises the existence of abundance of personal enthusiasm, of educated interest, and of noble individual effort, as manifested in societies which teach domestic hygiene, which promote wholesome cooking and the better feeding of children, which provide gymnasia and recreation grounds, which organise cadet corps for boys and healthy exercise for girls, which advocate sanitary houses with sufficient dwelling space for the people. All these (it is said) would be more strongly supported by the existence of a national organisation ready to encourage any effort, and to support well-considered legislative or executive action on behalf of the physical welfare of our fellow subjects.

We confess that this programme fills us with a good deal of doubt as to the actual practicability of its proposals. There is, we believe, much opportunity for good national work in the way of diffusing knowledge by lectures and publications, as well as by skilled inquiry into the actual state of given populations, followed by reports and meetings for the purpose of bringing home the facts thus disclosed to the minds and consciences of the local ratepayers and local authorities who are mainly responsible for them. A National Society might well undertake work of this description, and, if it were sufficiently well supported, it might assist many local or special societies by grants in aid of their proceedings. But we greatly doubt whether the local societies, speaking generally, would be as willing to accept counsel or control as they probably would be to accept money. Ladies and gentlemen engaged in "noble individual effort" would be likely to think themselves quite capable of determining, without outside help, the forms which their noble efforts should assume, and the channels into which they should be directed. They would be likely to declare that they were the best judges of the requirements of their own district, and of the precise manner in which these requirements could most advantageously be supplied; and they would protest against any hard and fast conditions emanating from a central board or committee. Such a committee, on the other hand, human nature being what it is, would be more than likely to base a claim to superior wisdom upon the wide extent of its experience, and it might even, under conceivable conditions, become tiresome or exacting in its requirements. The relations between the Local Government Board and Boards of Guardians, although defined by law and not admitting of dispute, have not always been entirely harmonious; and it is certain that the scheme described in the words we have quoted—the scheme for harmonising scattered individual effort—although it might be rendered successful by the consummate tact of some single individual, would almost invite difficulties when administered by an average human committee. We are much mistaken if the executive of the League will not find it necessary to be somewhat more precise than they have been hitherto, before they will secure such an amount of support as will enable them to accomplish any really national work. We have the greatest possible sympathy with their objects, but must confess to a wish for more light as to the means by which these objects are to be attained.

